# Neutrophils in glioma microenvironment: from immune function to immunotherapy

**DOI:** 10.3389/fimmu.2024.1393173

**Published:** 2024-05-08

**Authors:** Chao Sun, Siwen Wang, Zhen Ma, Jinghuan Zhou, Zilin Ding, Guoqiang Yuan, Yawen Pan

**Affiliations:** ^1^ The Second Clinical Medical School, Lanzhou University, Lanzhou, China; ^2^ Department of Neurosurgery, Lanzhou University Second Hospital, Lanzhou, China; ^3^ Key Laboratory of Neurology of Gansu Province, Lanzhou University Second Hospital, Lanzhou, China

**Keywords:** glioma, neutrophil, tumor-associated neutrophils, immunotherapy, tumor microenvironment, neutrophil extracellular trap

## Abstract

Glioma is a malignant tumor of the central nervous system (CNS). Currently, effective treatment options for gliomas are still lacking. Neutrophils, as an important member of the tumor microenvironment (TME), are widely distributed in circulation. Recently, the discovery of cranial-meningeal channels and intracranial lymphatic vessels has provided new insights into the origins of neutrophils in the CNS. Neutrophils in the brain may originate more from the skull and adjacent vertebral bone marrow. They cross the blood-brain barrier (BBB) under the action of chemokines and enter the brain parenchyma, subsequently migrating to the glioma TME and undergoing phenotypic changes upon contact with tumor cells. Under glycolytic metabolism model, neutrophils show complex and dual functions in different stages of cancer progression, including participation in the malignant progression, immune suppression, and anti-tumor effects of gliomas. Additionally, neutrophils in the TME interact with other immune cells, playing a crucial role in cancer immunotherapy. Targeting neutrophils may be a novel generation of immunotherapy and improve the efficacy of cancer treatments. This article reviews the molecular mechanisms of neutrophils infiltrating the central nervous system from the external environment, detailing the origin, functions, classifications, and targeted therapies of neutrophils in the context of glioma.

## 1 Introduction

Glioma, with insidious onset and terrible prognosis, is the most frequent aggressive primary brain tumor in adults. Glioblastoma (GBM) as the grade 4 glioma comprises only IDH wild-type (wt) tumors and is the most notoriously hard to treat. The standard of care (SOC) for GBM involves a combination of maximal surgical resection, as well as concurrent administration of temozolomide (TMZ) chemotherapy and radiotherapy (1). But it is still ineffective in preventing relapse or progress of GBM. Recently, the discovery of the cranial bone-dura mater channels and intracranial lymphatic vessels has brought significant attention to the role of immune cells in brain tumors. Immunotherapies targeting the TME may become a novel treatment.

Glioma TME is a complex system that undergoes dynamic changes based on genetic and epigenetic alterations in gliomas. The glioma TME consists of a variety of non-tumor cells in addition to tumor cells, mainly including immune cells, vascular cells, stromal cells, and extracellular matrix (2, 3). These constituents engage in intimate interaction which is crucial to the development of tumors. In the immune cell population of the TME, innate immune cells are predominant, primarily comprising Tumor-Associated Macrophages (TAMs), Tumor-Associated Neutrophils (TANs), Natural Killer (NK) cells, and Myeloid-Derived Suppressor Cells (MDSCs). Among these, TAMs can constitute 30-50% of the cellular composition in gliomas, leading researchers to concentrate more on macrophages that dominate the TME and to pay insufficient attention to neutrophils in gliomas. The ratio of mononuclear cells to neutrophils in GBM is approximately 1:7 to 1:10, which is opposite to their ratio of 7:1 in circulation (4). Neutrophils are incapable of proliferation and possess a highly contested lifespan that varies from 19 hours to 5.4 days (5–7). The rarity of neutrophils within the central nervous system, alongside their uncertain lifespan, leads to the underestimation of their role in cancer. However, with the deepening of glioma research, neutrophils have been discovered to be crucial in glioma. Increasing evidence shows that the ratio of neutrophils to lymphocytes (NLR) in glioma patients has diagnostic and prognostic value (8). Neutrophils play a significant role in anti-bacteria and inflammation via phagocytosis, degranulation, neutrophil extracellular traps (NETs) release, and antigen presentation (9, 10). Moreover, similar to the polarization states of macrophages M1/M2, there is also a classification method of N1/N2 in neutrophils, which respectively play anti-tumor and pro-tumor roles in the TME. They show complex and dual functions in different stages of cancer progression (11–13). Various chemokines and their receptors mediate the migration of neutrophils from the periphery to the brain parenchyma. They contribute to carcinogenesis by promoting tumor proliferation, invasion, migration, angiogenesis, immunosuppression, and enhancing tumor drug resistance (14).Targeting TANs may serve as a novel generation of immunotherapy and enhance the beneficial effects of cancer therapies (15).

## 2 The development, recruitment and migration of neutrophils

**Figure 1:**
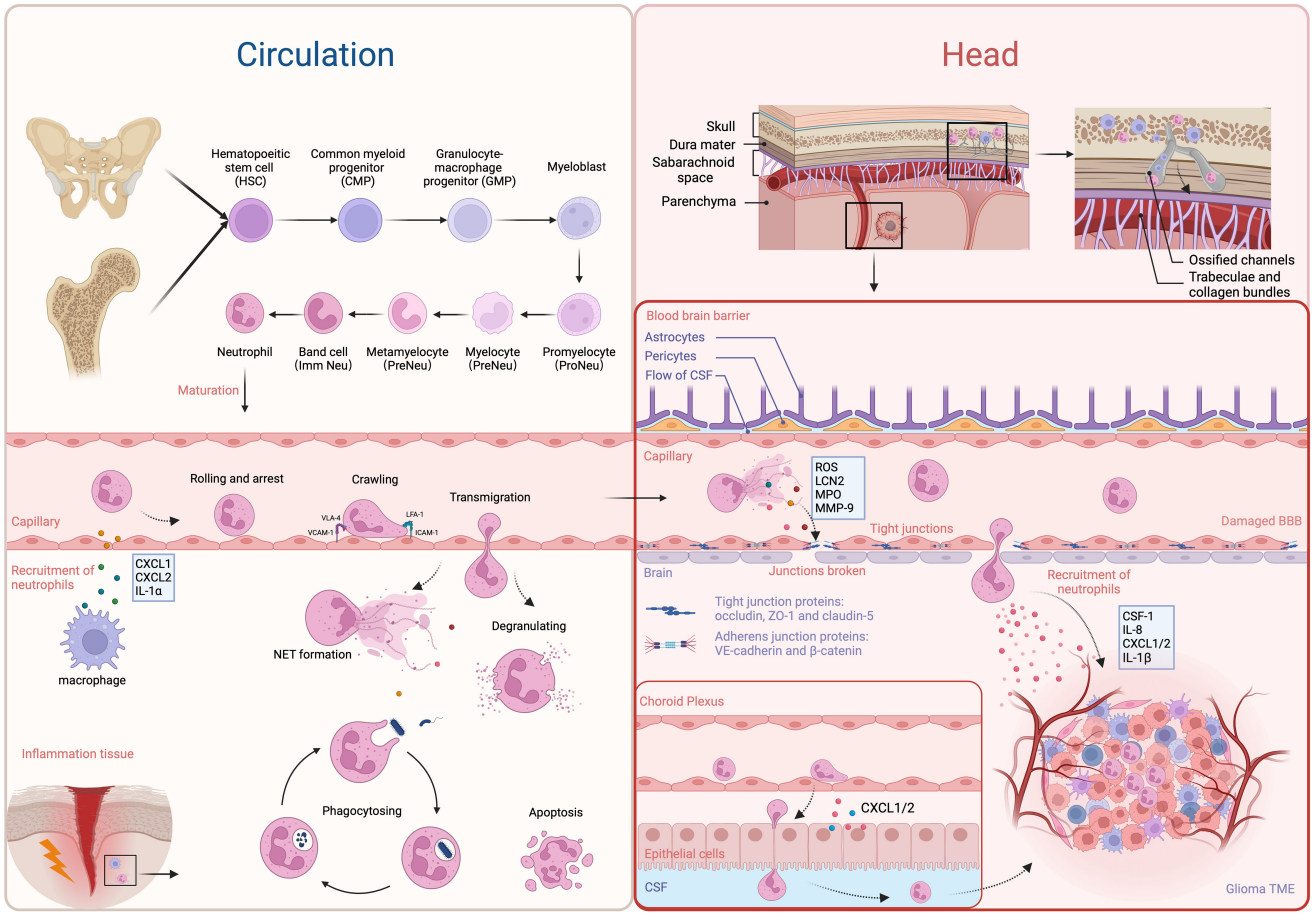
The development, recruitment and migration of neutrophils. The most majority of neutrophils in circulation are derived from bone marrow in long bones and the ilium. Starting from HSCs, they undergo several steps of development to become mature neutrophils, which are then released into the circulation. The resident macrophages and epithelial cells secrete chemoattractants to attract neutrophils (e.g. CXCL1, CXCL2, and IL-1α). Through the receptors LFA-4, ICAM-1, VCAM-1 and VLA-4, they migrate to sites of inflammation and exert their anti-bacteria and pathogen-clearing effects. Neutrophils in the brain primarily originate from bone marrow in the skull and adjacent vertebrae, with a smaller proportion derived from circulation. They only cross the BBB or choroid plexus and enter the brain parenchyma under pathological conditions, attracted by chemotactic factors in the TME. Furthermore, factors (e.g. ROS, LCN2, MPO, and MMP9) released by NETs can disrupt the integrity of the BBB, facilitating the infiltration of neutrophils into the brain parenchyma. On the other side, neutrophils also infiltrate the TME through chP under the attraction of CXCL1/2. In the TME, TANs are predominantly found in the central necrotic zone, while TAMs tend to accumulate at the periphery of the tumor. Created with BioRender.com.

The most common kind of innate immune cell in human beings are neutrophils. They make up about 70% of all white blood cells in circulation and are the most frequent type of white blood cells, acting as the frontline of immune system protection against bacterial and fungal diseases (16). The understanding of the origin of neutrophils has been evolving. It is well known that white blood cells originate from red bone marrow. In infants and young children, most of their bone marrow consists of red marrow. As age increases, some areas of red marrow undergo fatty transformation and become yellow marrow. Therefore, adult red marrow is mainly found in the medullary cavities of long bones (such as the humerus and femur) and the sparse bony trabeculae in flat bones (such as the ilium). Previous views suggested that immune cells in the circulation enter the cerebrospinal fluid and meninges, serving as a source of immune cells in that region. Whereas, some evidence indicates that red marrow can also be found in the skull, and the infiltration of white blood cells in the brain is more likely coming from the skull (**Figure 1**). Exploring the cranial bone cavity may provide a new direction for immunotherapy of intracranial tumors. It has been reported that some white blood cells are released into the circulation from the bone marrow through the nasal sinuses (17). As research deepens, researchers have discovered microscopic channels between the cranial bone marrow cavity and the dura mater through studies on mouse skulls and human craniectomy specimens. Neutrophils migrate to the brain through these “shortcuts.” Furthermore, it has been observed that the cranial bone marrow contributes significantly more neutrophils to the brain than the tibial bone marrow (18). In 2021, Andrea Cugurra et al. demonstrated that neutrophils originated from cranial and vertebral bone marrow can migrate to the meninges and parenchyma via ossified channels containing blood vessels (19, 20). In 2023, Meeki Ld et al. further confirmed the recruitment of TANs from the skull in GBM (21). They proposed that the role of the skull in supplying immune cells to the GBM TME is pronounced, and that systemic bone marrow sites could not adequately compensate for the absence of skull marrow. In addition, there are lymphatic pathways parallel to the dural venous sinuses, which directly connect the brain to deep cervical lymph nodes (22, 23). These lymphatic channels may contain dendritic cells and neutrophil nests associated with antigen presentation in the peripheral immune system, making them potential targets for future treatments (24, 25). The extent to which these pathways are involved in brain tumors is still unclear.

The granulocyte-monocyte progenitors (GMPs) are produced by hematopoietic stem cells (HSCs) in the bone marrow. GMPs are considered to be the upstream precursor cells for all neutrophils in hematopoiesis. Regulated by granulocyte colony-stimulating factor (G-CSF), GMPs subsequently undergo several stages, including myeloblasts, promyelocytes, myelocytes, metamyelocytes, and band cells, finally developing into mature neutrophils (26). Neutrophils generally release more from the bone marrow and enter the circulation when functional CXC chemokine receptor 4 (CXCR4) gets lost and CXCR2 is expressed on neutrophils (27, 28). During emergency granulopoiesis, band cells complete maturation in injured tissue (29). The recruitment of neutrophils involves interactions between them and endothelial cells. During tissue damage or infection, neutrophils undergo a series of actions, including rolling, arrest, crawling, and transmigration, ultimately crossing the endothelial cells to enter inflammatory tissue under the influence of chemoattractants secreted by resident macrophages and epithelial cells, such as CXC-chemokine ligand 1 (CXCL1), CXCL2, and interleukin 1α (IL-1α) (30, 31). Endothelial intercellular adhesion molecule-1 (ICAM-1) and vascular adhesion molecule-1 (VCAM-1) regulate the arrest phase, respectively, through linking to neutrophil integrins lymphocyte functional antigen-1 (LFA-1) and very late antigen-4 (VLA-4) (31). Neutrophils then eliminate invading microorganisms through phagocytosis, degranulation (releasing granules containing cytotoxic substances), or in the form of NETs by releasing DNA-microbial protein complexes (32).

Under normal physiological conditions, immune cells are rarely found in the brain parenchyma. Those cells are mostly found in the organs surrounding the brain ventricles and choroid plexus, the meninges, and the perivascular space (33). The blood-brain barrier (BBB) and blood-cerebrospinal fluid barrier (BCSFB) constitute unique structures in the brain, which hinder the entry of immune cells and molecules into the brain, leading to immune responses different from the peripheral system (34). The BBB is an intricate system primarily formed by endothelial cells tightly connected through junctional molecules, astrocytic end-feet, and pericytes. Through tight junction (TJ), adherens junctions (AJ), and gap junctions (GJ) proteins, BBB restricts serum molecules and blood cells to entering the brain, and maintains the stability of the intracranial environment (35). Molecules such as occludin, claudin-5, and zonula occluden-1 (ZO-1) play crucial roles in regulating BBB permeability. Claudins are key factors in maintaining the “tightness” of the seal between adjacent endothelial cells (ECs) (36). Specifically, claudin-5 is the dominant transmembrane TJ protein on the BBB, maintaining vascular stability and regulating free molecular exchange (37). Furthermore, the BCSFB is responsible for isolating blood from cerebrospinal fluid and is frequently linked to the arachnoid mater and choroid plexus (ChP) (38). The presence of junctional molecules in the epithelial lining of the BCSFB at the choroid plexus allows for the selective permeability of blood components. However, due to its low expression of claudin-5, it is more permeable to small/large molecules and immune cells (39, 40). Neutrophils migrate into the brain under the attraction of CXCL1/2 chemokines secreted by the ChP epithelium in traumatic brain injury (TBI) models (41). Similarly, in the model of stroke, neutrophils also infiltrate the brain through the ChP (42). Although the BBB and BCSFB share functional similarities, more research has been conducted on the BBB due to its involvement in central nervous system pathology (31).

Inflammation and tumors share some common mechanisms for recruiting neutrophils. Research has shown that numerous cytokines and chemokines are associated with neutrophil recruitment, such as G-CSF, IL-1β, CXCL1, CXCL2, CC-chemokine ligand 3 (CCL3), and ICAM1 (43). G-CSF, in particular, is a major cytokine that regulates neutrophil recruitment and development (44, 45). Besides G-CSF, other factors that can enhance neutrophil proliferation include stem cell factor, IL-6, and granulocyte-macrophage colony-stimulating factor (GM-CSF) (46–48). The GBM-expressed long non-coding RNA (lncRNA) LINC01116 interacts with the transcriptional regulator DDX5, enhancing the transcriptional expression of IL-1β, thereby promoting the recruitment of neutrophils (49). DDX5 interacts with NF-kB p50, increasing the expression of p50 and promoting the growth of gliomas (50). Additionally, high expression of CD133 in gliomas facilitates the recruitment of neutrophils by modulating the regulation of IL-1β and its downstream chemotactic factors (51). Furthermore, the number of neutrophils around the necrotic areas is notably higher. Mesenchymal (MES) cellular state could be induced in glioma stem cells (GSCs) by IL-1β, which may also greatly improve the self-renewal capacity of GSCs (52, 53). Furthermore, it has been demonstrated that a number of other cancer cell-derived factors, including IL-8, CXCL3, CXCL5, and osteopontin (OPN), are efficient neutrophil chemoattractants (54, 55). IL-8 is produced by GBM via various pathways that can be induced by FasL and high mobility group protein 1 (HMGB1) (54, 56). It was first discovered that neutrophils expressed the chemokine receptor CXCR2, which is the receptor for IL-8 (57). CXCR2 was found to be expressed in GBM and significantly associated with tumor recurrence (58). Subsequently, it was found that CXCR2 also serves as a common chemokine receptor for CXCL3 and CXCL5. OPN is highly expressed in the necrotic areas of gliomas under the influence of hypoxia-inducible factor 1α (HIF-1α). OPN promotes the migration of neutrophils *in vitro* and co-localizes with them (55). These findings indicate that neutrophils are recruited to necrotic areas under the influence of OPN, CD133, and IL-1β. Neutrophils are mainly restricted to the necrotic core, which is the central area of the TME. Moreover, Patricia P. Ye et al. have shown that neutrophils are attracted to tissue damage sites by certain tumoral insults, such as ischemia that occurs in the early phases of tumor progression (59). This recruitment establishes a positive feedback loop that significantly amplifies necrosis development in GBM. TANs are associated with necrosis in the TME spatially and temporally. TANs correlate with the extent of tumor necrosis positively and predict lower survival rates in GBM patients (59). Monocytes and macrophages, are located around blood vessels and the peri necrotic area, while most microglia are found at the invasive edge of the tumor (60, 61).

Activated astrocytes and microglia release pro-inflammatory cytokines that upregulate adhesion molecules such as ICAM-1 on brain microvascular endothelial cells (BMECs), promoting neutrophil adhesion (62). Binding of neutrophil integrins and other stimuli leads to NETs forming and inflammatory chemicals releasing, containing reactive oxygen species (ROS), matrix metalloproteinase-9 (MMP-9), myeloperoxidase (MPO), and lipocalin-2 (LCN2). These neutrophil-derived substances help break down the BBB, increasing permeability, and reducing levels of tight junction proteins (claudin-5, occludin, and ZO-1) (35, 63). Therefore, neutrophils are more easily able to enter the brain parenchyma through the damaged BBB. The current understanding of the mechanisms underlying neutrophil recruitment in GBM is still limited. Further experiments are needed to explore precise mechanisms of signal transduction.

## 3 Neutrophil heterogeneity and plasticity

Different terms are used to classify neutrophils, such as N1/N2 neutrophils, TANs, and polymorphonuclear myeloid-derived suppressor cells (PMN-MDSCs) (64, 65). Mature neutrophils express specific cell surface proteins, including CD14^-^, CD15^+^, CD66b^+^, and CD16^+^ (66). Interestingly, the surface markers of PMN-MDSCs are similar to neutrophils,which includes CD14^-^, CD15^+^, CD66b^+^,CD11b^+^, CD33^+^, and HLA-DR^-^ (67, 68). They both have similar morphology and phenotype, so some studies equate the two. However, they have some functional differences (65). Both PMN-MDSCs and mature neutrophils are capable of suppressing immune responses, but neutrophils are limited to cell activation. PMN-MDSC overlaps functionally with neutrophils and actually describes a subset of neutrophils (69). To distinguish them, these neutrophils are commonly named immunosuppressive neutrophils. The similarities and differences between immunosuppressive neutrophils and PMN-MDSCs are discussed in detail in the review by Benedict et al. (69).

Fridlender et al. (2009) categorized anti-tumor and pro-tumor TAN types, designating them as N1 (anti-tuomr) and N2 (pro-tumor), respectively (70). Interferon-beta (IFN-β) and transforming growth factor-beta (TGF-β) are responsible for polarizing neutrophils into N1 and N2, respectively (71, 72). The N1/N2 concept has now evolved to encompass broader aspects including neutrophil phenotypes, specialized morphologies, and particular functions. N1 neutrophils exhibit enhanced antigen presentation, greater phagocytic activity, stronger cytotoxicity, and an increased production of cytokines beneficial for antitumor immune responses (69). Conversely, N2 neutrophils show the opposite traits. Generally, N1 neutrophil has a hypersegmented nucleus, while N2 neutrophil has a circular nucleus. The N1 neutrophil phenotype is associated with high expression of Fas, TNF-α, and ICAM-1, while N2 expresses CXCR4, arginase1 (ARG1), VEGF (vascular endothelial growth factor), and MMP-9. Depletion of N1 was found to promote tumor growth (70, 73). Although there are functional differences, surface markers to distinguish N1 and N2 TAN have not yet been identified. Currently, the cellular state of neutrophils can be indirectly determined based on the expression of relevant genes and molecules (**Table 1**). The cellular state is uncertain, and the changes described above should only be interpreted as indicative of a general tendency. They provide a benchmark for reference but do not definitively determine that N1/N2 will invariably follow this pattern. Nonetheless, knowledge about neutrophil subgroups is still incomplete and controversial, and there is still a lack of specific molecular markers, unified research methods, and authoritative expertise.

**Table 1:** The characteristics of N1/N2 neutrophils.

Type	Stimulatory Cytokines	Morphology	Related genes expression	Related secretions	Functions	Reference
N1	IFN-β	Hypersegmented nucleus	Increased ICAM1, VCAM1, CD95/Fas, TNFR, and IL-1R	Increased IFN-β, TNF-α, IP-10, IL-22, and IL-12a	Anti-tumor/inflammatory: enhanced antigen presentation, phagocytosis, cytotoxicity, anti-inflammatory cytokines, toxin and ROS; promotion of tumor cell apoptosis	(32, 70, 74–77)
N2	TGF-β	Circular nucleus	Increased TGF-β, ARG1/2, gelatinase, iNOS, CXCR4, CD184, VEGF, Bv8, and S100A8/9	Increased IL-1β, CCL4, CCL5, IL-6, IL-8 and IL-10	Pro-tumor/inflammatory: support angiogenesis, tumor cell proliferation and invasion, NETs formation, immunosuppression, secrete chemokines, cytokines and ROS/RNS	(32, 70, 74–79)

TNF-α, Tumor necrosis factor alpha; IL-1, interleukin-1; IP-10, interferon gamma-induced protein 10; S100A8/9, S100 calcium-binding protein A8/9; iNOS, inducible nitric oxide synthase; VEGF, vascular endothelial growth factor; CCL4, C-C motif chemokine ligand 4.

Based on density, neutrophils can be further subdivided into three groups: immature low-density neutrophils (LDN), mature LDN, and mature high-density neutrophils (HDN) (80–82). Immature LDN has low levels of CD16, CD11b, and high CXCR2 expression (81). The phenotype of normal or HDN is similar to that of anti-tumor N1, while the LDN show impaired function and immunosuppressive characteristics (80, 81, 83). HDN can switch to LDN depending on factors in the TME. Theoretically, there is a middle state, the N0 state. It lies between N1 and N2, which has a neutral effect on tumors. One study indicated that IFN-β might decrease the pro-tumor function of N2 instead of driving the N2-N1 switch (71). This is similar with the MDSC-like and tumor-promoting activities frequently observed in TAN in the B16 model (84–86). Therefore, “N1-N2” refers to a continuum of distinct neutrophil activities, and anti-tumor polarization may be understood as a decrease in activity that promotes tumor growth.

## 4 Functions of neutrophils after activation

Neutrophil activation occurs prior to their infiltration (87).Under the stimulus or influence of chemokines and cytokines, neutrophils priming happens to get ready to immune response (88). Following activation, they participate in immune regulation through mechanisms such as NET formation, degranulation, and phagocytosis (9).

### 4.1 NET and NETosis in neutrophils

NET formation is a unique function of neutrophils. Several granular proteins and molecules are involved in the process of NET formation (89). Initially, the released MPO, cathepsin G, and NE from neutrophils enter the cytosol, where they begin to breakdown the nuclear lamina (90–92). Subsequently, peptidyl-arginine deaminase 4 (PAD4) gets into the nucleus and induces citrullination of histones, resulting in chromatin decondensation (93). After passing through the cellular and nuclear membranes, chromatin, along with its associated granular proteins, is ultimately secreted into the extracellular space. NETs promote the occurrence and invasion of GBM by expressing NET-related proteins (including elastase, protease-3, and protease G) (94–96). Neutrophil-derived NETs in cancer can act as a physical blockade, preventing immune cell infiltration and restricting the interaction between T cells and cancer cells (97). Certain proteins within the NETs can cause an immunosuppressive effect. An experiment revealed that the mesh-like structure of NETs is modified by HMGB1 (54). The externalized HMGB1 interacts with receptor for advanced glycation end products (RAGE) on GBM cells, resulting in the activation of the transcription factor NF-κB, which in turn enhances the expression and secretion of IL-8. The released IL-8 interacts with CXCR2 on neutrophils, triggering the generation of ROS and additional NET formation. This creates a positive feedback loop that promotes the progression of GBM (54).

Neutrophill cell death can be caused by NET formation, but this is not always the case. This regulated cell death that depends on NETs is called NETosis (98). NETosis occurs in two forms: suicidal and vital NETosis (99). Suicidal NETosis occurs in the presence of NADPH oxidase activity, leading to the release of neutrophil extracellular traps (NETs) and concurrent neutrophil death. In contrast, vital NETosis is characterized by the survival of neutrophils post-NET release, with intact nuclear and plasma membranes that retain inflammatory functions. The mechanisms underlying NETosis have been extensively discussed in other reviews (98, 100). NETosis is not limited to neutrophils. Cell direct interaction and receptor activation appear to be the catalysts for NETosis (99, 101, 102). NETosis also occurs in neutrophils within brain parenchyma. Subsequent neuronal injury and microglial activation amplify neuroinflammation and lead to neuronal loss (103). NETosis has shown great potential and may have important implications for future therapeutic and preventive strategies (104).

### 4.2 Degranulation in neutrophils

Degranulation is the process by which the contents of neutrophil granules are secreted into the extracellular space via exocytosis (105). Degranulation plays a role in various stages of neutrophil function. Primary (azurophilic granules), secondary (specific granules, tertiary (gelatinase), and secretory vesicles are the four main granule types found in neutrophils (87). Degranulation typically involves the secretion of contents from the first two types of granules, while the granules of the latter two types are often secreted periodically (106). Similar to exocytosis, degranulation is regulated by small Ras associated binding protein (Rab) GTPases, particularly the Rab27/Munc13-4 pathway (107).

### 4.3 Phagocytosis in neutrophils

Phagocytosis is another crucial function of neutrophils, responsible for effectively eliminating pathogens and/or debris (108, 109). However, the discovery of the phagocytic function of neutrophils in neuroscience has been relatively lagging. Phagocytic activity of cell debris is often thought to be caused by microglia in the CNS or macrophages in the peripheral nervous system (PNS) (110). Microglia and macrophages share a similar phagocytic function with neutrophils. To internalize different pathogens, neutrophils with fragment crystallizable (Fc) γ receptors (FcγRI, FcγRII, and FcγRIII) interact with IgG and other complement-mediated particles (111). This process results in the formation of phagosomes, which subsequently merge with lysosomes, generating an acidic environment for the enzymatic degradation of contents (112, 113).

## 5 Roles of neutrophils in glioma immunity

Immune infiltration is a characteristic of chronic inflammation, which can cause tissue damage and ultimately lead to tumor progression (64). Neutrophil infiltration begins early in tumorigenesis and persists throughout tumor progression (114). Like two sides of the same coin, neutrophils exhibit a complex dual function in different stages of cancer progression they possess an anti- or pro-tumoral phenotype through various molecular mechanisms of interaction with tumor cells, as well as modulating the development of tumor cells by regulating other immune cells (115, 116).

### 5.1 Glioma-neutrophils crosstalk

Multiple studies are currently exploring the correlation between neutrophils and cancer. Tumor cells secrete G-CSF, which promotes the proliferation of myeloid cells, resulting in a rise of neutrophils and an elevation of the neutrophil-lymphocyte ratio (NLR) in patients with GBM (117–119). In many types of tumors, NLR has emerged as a prognostic factor for predicting survival, such as colorectal cancer, liver cancer, breast tumor, and GBM (8, 120–122). In GBM patients undergoing radiotherapy or chemotherapy, an increase in neutrophils is associated with higher tumor grades and poor prognosis (123, 124). An NLR>4 is associated with poor prognosis, while an NLR<4 is linked to a better prognosis in GBM patients with wild-type IDH1 (125). In a zebrafish GBM model, neutrophils were observed to be recruited early in the tumorigenesis process, and their presence increased tumor cell proliferation due to the release of ROS, that could lead to DNA damage and trigger tumorigenesis in surrounding cells (126). Another *in vivo* experiment demonstrated that a higher percentage of Ki-67-positive cells (an antigen that marks cell proliferation status) in GBM is related to a higher concentration of neutrophils in peripheral blood. A high concentration of neutrophils appears to promote GBM proliferation and is more pronounced in high-grade GBM (127). Compared with non-GBM tumors, the Ki-67 positivity rate in high-grade GBM increases nearly two-fold, consistent with a higher NLR (>3).

Research on neutrophils in glioma is still in its early stages. In-depth study of the inherent characteristics of tumor cells, including genetic and epigenetic changes, can help explore the role of neutrophils in the TME (128). The activation of K-ras and the loss of Tp53 in pancreatic tumors promote the release of CXCR2 ligands (129), while PTEN and TP53 deficiency in prostate cancer lead to the release of CXCL17, both of them enhance neutrophil recruitment and contribute to TME immune suppression (130). In gliomas, genetic backgrounds and molecular states are correlated with neutrophil infiltration. For instance, IDH mutations are a favorable prognostic indicator for glioma patients, and neutrophil infiltration is less common in IDH-mutated gliomas compared to wild-type GBMs due to suppression of genes linked to chemotaxis and lack of immunosuppressive effects (131–133). TERT mutations promote neutrophil infiltration (134). GBM is categorized into three subtypes by the Cancer Genome Atlas (TCGA) initiative: mesenchymal (MES) subtype indicated by NF1 and PTEN loss; proneural (PN) subtype linked to PDGFRA amplification/mutation or CDKN2A homozygous deletion; and classical (CL) subtype defined by EGFR amplification/mutation (135, 136). In mouse models, tumors with NF1 downregulation infiltrate more neutrophils and microglia but less monocytes (137). A research about GBM heterogeneity suggested that MES GBM subtype is mainly composed of MES-like cells with NF1 loss (138). Furthermore, MES GBMs have more necrosis and increased number of macrophages and neutrophils than other TCGA subtypes (135, 139, 140). The mechanisms responsible for their recruitment remain to be elucidated (136).

Neutrophils are thought to conduct their anti-tumor effects through many ways. Neutrophils have the ability to suppress the growth of early-stage tumors (141–143). Neutrophils may eliminate tumor cells by directly contacting them and producing ROS (70, 143, 144). Specifically, the H_2_O_2_ secreted by neutrophils induces lethal Ca2+ influx mediated by TRMP2 channels, ultimately killing tumor cells. Additionally, a mechanism involving tumor cell apoptosis mediated by Fas ligand/Fas interaction has been discovered (145). Hepatocyte growth factor (HGF) present in the TME induces the recruitment of neutrophils and the production of nitric oxide (NO) (116). In gliomas, superphysiologic levels of NO result in the killing of tumor cells (146). Neutrophils can exert anti-tumor effects through a recognized mechanism called antibody-dependent cellular cytotoxicity (ADCC). Studies have shown that the receptors involved in neutrophil ADCC include Fc and Mac-1 (147, 148). Extensive literature suggests that the FcγRIIa receptor plays a major role in ADCC and shows polymorphic variants in different tumors (148, 149). Monoclonal antibodies bind to activating Fc receptors on the surface of neutrophils to initiate indirect-mediated cell death. The precise mechanism of cell death mediated by ADCC remains unclear and may be associated with “trogoptosis”. Neutrophils take a “bite” out of the cancer cell membrane, causing membrane damage and resulting in necrotic cell death (150). Neutrophils also show antitumor activity through TNF related apoptosis-inducing ligand (TRAIL), binding to the TRAIL receptors on tumor cells to induce cytotoxicity (151).

As previously mentioned, neutrophils are scarce in brain parenchyma; however, gliomas release various chemokines and cytokines (e.g., CSF-1, IL-8, IL-1β, CXCL1/2) that facilitate the recruitment of neutrophils within the TME.Cancer cells modulate the ratio of pro-tumor and anti-tumor neutrophils depending on the context (80). The depletion of neutrophils in the early stages of tumor development suggests a compromised anti-tumor capacity as the tumor progresses. Mariana R. Aubin et al. demonstrated that neutrophils attack tumor cells and reduce their viability within the first 24 hours of contact with GBM cells (152). With prolonged contact, tumor cells successfully reprogram the functionality of neutrophils into a pro-tumor phenotype, highlighting the heterogeneity of tumor-infiltrating neutrophils depending on their actual time within the tumor microenvironment. Notably, TANs associated with brain tumors show significant differences compared to neutrophils in the circulation. In syngeneic mouse models, neutrophils from healthy mice inhibit the tumorigenicity of GSCs, whereas neutrophils from tumor-bearing mice promote tumor progression and immune suppression (114). TANs have an extended lifespan, immune suppression, and pro-angiogenic potential (153). *In vitro* experiments have shown that IL-6 and IL-8 produced by glioma cells prolong the lifespan of neutrophils, indicating that neutrophils and glioma cells interact reciprocally (154). A study analyzing clinical samples from over 190 different human brain tumors confirmed the tissue-specific presence of neutrophils in brain tissue, showing a distinct inflammatory phenotype compared to primary brain lymphomas. TNF-α and ceruloplasmin (CP) are soluble inflammatory mediators that may be responsible for this inflammatory phenotype (153). The glioma TME inhibits the production of ROS in neutrophils and facilitates their polarization from an antitumoral N1 phenotype to a protumoral N2 phenotype. However, the precise mechanisms affecting neutrophil phenotypic changes within the TME remain unclear (153).

Neutrophils promote proliferation, angiogenesis, and invasion of tumor cells through various mechanisms (155). There have been reports that neutrophils release factors that promote GBM growth by increasing oxidative stress and releasing NET (94). HMGB1 released by NET can activate the TLR9 de-peptide pathway, thereby maintaining tumor cell growth (156). By cleaving laminin-111, neutrophil-secreted MMP9 and neutrophil elastase (NE) activate integrin signaling, which in turn promotes the growth of cancer cells (157). Histones in NETs disrupt the adhesion and tight junctions of vascular endothelial cells, while MMP-9 degrades the basement membrane of type IV collagen (103). Both actions increase the permeability of the BBB, making it easier for neutrophils to enter the TME and thereby promote tumor progression. NE contributes to the destruction of brain tissue and facilitates glioma invasion (158). Roeltje R. Maas and colleagues’ research indicated that in gliomas, TANs showed higher expression of various pro-angiogenic genes (such as VEGFA, THBD, and ICAM1). Compared to neutrophils in peripheral blood, TANs were enriched with the angiogenesis-related factor S100A9 and MMP9 (153). Neutrophil-released BV8, S100A8/9, and MMP9 are key factors in activating VEGF-A, thereby promoting angiogenesis (159). Furthermore, Neutrophils can induce drug resistance in gliomas. Anti-angiogenic therapy (e.g., bevacizumab) increases neutrophil infiltration, which activates the S100A4 signaling pathway, upregulating GSC self-renewal and mesenchymal transition. This leads to acquired drug resistance in tumor cells, thereby diminishing the efficacy of the treatment (160). Similarly, in animal models, Ly6G+ neutrophils and myeloid-derived suppressor cells (MDSCs) promote the transformation of cancer cells into GSCs through the regulation of the NOS2-NO-ID4 signaling axis (161).

### 5.2 Neutrophils-other immune cells crosstalk

The crosstalk between neutrophils and other immune cells is also complex, and their functions are not singular. TAMs and tumor-infiltrating lymphocytes (TILs) are key elements in the TME. By modulating the recruitment, characteristics, and phenotypes of these immune cells, neutrophils can have an impact on tumor growth. Neutrophils attract T cells and other leukocytes by producing chemokines, such as CCL2, CCL3, CXCL1, CXCL2, and CXCL10 (141, 162, 163). Neutrophils participate in T cell-dependent anti-tumor immune networks. They express ARG1, ROS, and NO when G-CSF and TGFβ exist in the TME, which inactivates T cells (164). In the presence of GM-CSF and IFNγ, neutrophils tend to mature and acquire antigen-presenting cell (APC) functions, and they can stimulate T cell proliferation through interactions with relevant ligands (141, 142). The binding of galectin-9 from neutrophils to TIM3 on lymphocytes initiates a process that results in the death of T cells (165). TANs also induce apoptosis in unactivated CD8+ T cells through the production of NO and TNF-α (166). In breast cancer, IL17-producing γδ T cells transform neutrophils into immunosuppressive type to promote tumor cells metastasis (167). Neutrophils express immune checkpoint receptors PDL1 and VISTA. The direct contact between checkpoint ligands on neutrophils and immune checkpoints on T cells can hinder anti-tumor immune responses, potentially leading to T cell-mediated apoptosis or T cell exhaustion (168, 169). The suppression of T cells is associated with direct contact between the integrin Mac-1 on neutrophils and T cells (170). Furthermore, degranulated neutrophils in the peripheral blood of patients with GBM were found to be correlated with increased serum ARG1 levels and decreased expression of T-cell CD3ζ. These neutrophils can induce T cell suppression, and it could be reversed by inhibiting ARG1 or supplementing with arginine (171). It has been reported that H_2_O_2_ can inhibit T cell activation and proliferation by reducing NF-κB activation, inhibiting the TCR: CD3ζ chain, and suppressing cytokine production (26).

Additionally, neutrophils interact with macrophages and unconventional T cell (UTC) subsets, specifically CD4^-^CD8^-^TCRαβ^+^ double-negative UTCs (UTCαβ), which are crucial for effective anti-tumor immunity (172). Neutrophils enhance macrophage production of IL-12, promoting IFNγ generation and polarization of UTCαβ toward a type 1 immune response (172). In bladder cancer, NETs enhance the antitumor effect by increasing macrophage infiltration (173). Neutrophil-derived microvesicles (NDMVs) enhance the polarization of anti-inflammatory macrophages, while neutrophil-derived trails (NDTRs) induce the polarization of pro-inflammatory macrophages. Therefore, neutrophils exhibit a complex dual regulatory role within the TME with macrophages, requiring further experimental verification of the specific mechanisms involved (174).

In the crosstalk between neutrophils and DCs, prolonged contact with NETs leads to DC apoptosis, with NETs obstructing DC responses and suppressing antitumor immunity (175). Conversely, DCs secrete factors that activate DNase1L3, playing a role in NET degradation and thus hindering NET-induced recruitment of pro-tumorigenic neutrophils (176). Moreover, neutrophils induce DC maturation through TNF-α and ICAM3 and, via cell-cell contact, promote IL-12 production, ultimately driving T cell proliferation (177). In glioma TME, DCs are typically in an immature state, leading to reduced activation of effector T cells. Research has shown that overexpression of Nrf in DCs inhibits their maturation, but suppression of Nrf2 can restore DC activity (178). Activated DCs can secrete high levels of bioactive IL-12p70, enhancing NK cell activity and initiating CD8+ T cell immunity, thereby activating the collaborative antitumor capacity of immune cells (179). Interestingly, another study demonstrated the opposite result, where neutrophil activation in glioma patients correlated with elevated plasma IL12p70 levels, which is indicative of more aggressive glioma progression (180). The mechanisms of interaction between DCs and neutrophils in the glioma TME require further exploration. In mouse models, neutrophils can suppress NK cell activation in tumor-bearing mice, thereby inhibiting NK cell cytotoxicity, weakening antitumor effects, and enhancing tumor cell metastatic capabilities (181–184).

Finally, neutrophils influence the immunosuppressive environment by affecting the development of regulatory T cells (Tregs) (185). The reduced sensitivity of Tregs to oxidative stress in the TME leads to Treg enrichment, and neutrophils also recruit Tregs by secreting CCL17 (186–188). Additionally, a positive feedback loop mediated by the anti-inflammatory cytokine IL-10 between neutrophils and Tregs supports immunosuppression (189, 190).

## 6 Metabolism in neutrophils

**Figure 2:**
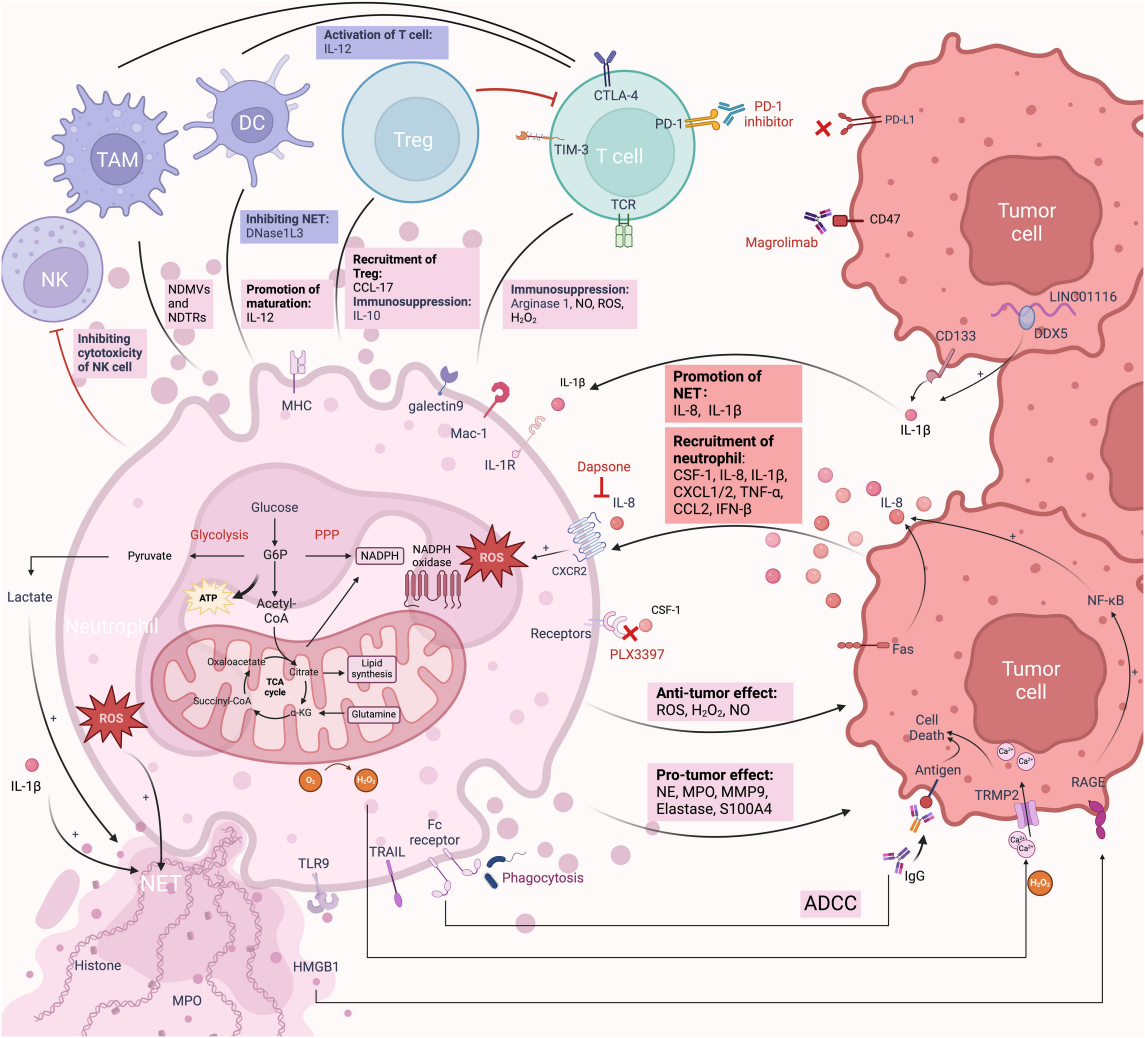
The immune responses of TANs in the TME. TANs and their interactions with tumor cells and immune cells constitute a complex TME. Tumor cells attract neutrophils into the TME through various chemokines and secretions. In the early stages of neutrophil entry into the TME, the secretion of NO, MPO, and ROS mediates tumor cell killing. Neutrophils also promote tumor cell death through ADCC and the secretion of H_2_O_2_. Over time, under the influence of chemokines and secretions in the TME, TANs transition from an anti-inflammatory to a pro-inflammatory phenotype. For example, IL-8 and IL-1β secreted by gliomas promote the formation of NETs through different mechanisms. ROS and lactate secreted by TANs also promote the formation of NETs. TANs associated secretion of NE, MMP9, elastase, and S100A4 plays crucial roles in tumor proliferation, invasion, and angiogenesis. Within the TME, TANs engage in complex interactions with TAMs, NK cells, DCs, T cells, and Tregs. Targeting these receptors is a form of neutrophil-mediated immunotherapy. Neutrophils themselves rely on glycolysis for energy supply and promote the generation of an acidic environment that favors tumor growth. Created with BioRender.com.

Similar to all other cells, metabolism is essential to the functioning of neutrophils (**Figure 2**). Neutrophils primarily rely on glycolysis and minimal mitochondrial respiration to regulate and maintain ATP production, which facilitates chemotaxis, phagocytosis, cytokine expression, ROS generation, degranulation, and NET formation in circulation and peripheral tissues (191). In response to stimulation, neutrophils switch from glycolysis to the mitochondria-mediated pentose phosphate pathway (PPP) to improve oxidative burst (192). PPP can generate NADPH, and then NADPH oxidase oxidizes it to produce ROS, which in turn causes the production of NETs. ROS generation in neutrophils is significantly influenced by mitochondrial activity. The high glycolytic potential of neutrophils contributes to lactate production in the TME. NET formation depends on lactate formation (191). This acidic environment supports tumor survival, progression, and inhibition of immune activity near tumor cells (14). When there is inflammation and local oxygen levels are low, the preference for glycolysis improves neutrophil survival rates (193). This partly explains why neutrophils tend to concentrate in hypoxic areas at the center of the TME. Mechanistically, activated mature neutrophils induce lipid peroxidation in tumor cells by transferring granules containing MPO, which increases lipid-based ROS in these cells. This process promotes ferroptosis and tumor necrosis, which indicates poor prognosis in GBM (59). During neutrophil oxidative bursts, MPO granules produce hypochlorous acid (HOCl), which is crucial for NETosis (194). The NETosis pathway requires the production of ROS and an increase in intracellular Ca2+ (195). This is mediated by mitochondrial ATP production, which maintains a positive feedback loop by activating purinergic receptors such as P2Y2 (196).

## 7 Neutrophils-targeted immunotherapy

Neutrophils-targeted therapeutic strategies can draw inspiration from macrophages, which also originate from myeloid cells. These strategies mainly involve inhibiting recruitment, reprogramming, and depleting neutrophils.

Due to the pro-tumor effects of neutrophils, blocking the recruitment of immunosuppressive neutrophils can help alleviate tumor progression to some extent. Given the powerful recruitment effects of G-CSF on myeloid cells, it is considered an ideal target for exploration (197). The anti-tumor therapy agent Pexidartinib (PLX3397) targeting CSF1R has completed a Phase II clinical trial for recurrent GBM (NCT01349036). Unfortunately, this treatment did not provide survival benefits to patients. In targeting IL-8/CXCR2-mediated neutrophil recruitment, Karpel-Massler et al. achieved certain efficacy by using dapsone to block IL-8-mediated neutrophil infiltration in gliomas (198, 199). A Phase I clinical trial (NCT03161431) investigating the use of CXCR2 inhibitor SX-682 for the treatment of melanoma cancer is currently recruiting participants.

Reprogramming neutrophils to the N1 phenotype and restoring their phagocytic function is also a promising strategy. TGFβ, as a polarization factor for N2 neutrophils, targeting its receptor presents a promising approach. The TGFβ receptor I kinase inhibitor Galunisertib (LY2157299) is currently under clinical trials in glioma patients. It is undergoing a Phase I clinical trial (NCT01682187) and a Phase II clinical trial (NCT01582269) for patients with recurrent GBM. Neutrophil-mediated tumor cell death is inhibited by CD47-SIRPα signaling (200). Targeting CD47-SIRPα can enhance the ability of neutrophils to eliminate tumors through ADCC (201). Currently, anti-CD47 and anti-SIRPα monoclonal antibodies (mAb) have shown preliminary efficacy in some solid tumors and have entered clinical trial stages, such as Hu5F9-G4 (anti-CD47, NCT02216409) and TTI-621 (anti-CD47, NCT02890368), and BI765063 (anti-SIRPα, NCT03990233). The anti-CD47 antibody Magrolimab, developed to target the CD47 receptor, has been tested in a Phase I clinical trial for glioma (NCT05169944). Anti-CD47 antibodies enhance the phagocytic activity of TAMs. In GBM mouse model, these antibodies show synergistic effects when combined with TMZ, PD1 antibodies, and activation of TLR3 and TLR9 (202–204). The involvement of neutrophils in this synergistic effect remains to be elucidated and could provide insights into therapeutic strategies. Exogenous administration of recombinant TRAIL or agonistic TRAIL-R antibodies can induce apoptosis in tumor cells and immunosuppressive cells (such as TAMs and Tregs), leading to an increase in cytotoxic T lymphocyte (CTL) numbers and enhanced phagocytic capabilities of neutrophils, monocytes, and macrophages (151).

Furthermore, reducing neutrophils by anti-Ly6G antibodies is a potential therapeutic strategy (59, 205). However, the use of anti-Ly6G antibody in experiments has not achieved consistent reduction in neutrophil levels, further research to explore more durable approaches is still needed. Inhibiting NET can weaken the pro-tumor effect of neutrophils. Experiments have found DNase I, PAD inhibitors, neonatal NET-inhibitory factor, metformin, and anti-HMGB antibodies are effective in reducing NET (206–210). The use of oncolytic herpes simplex virus (oHSV) for the treatment of glioma results in the upregulation of IGF2BP3 and increased NET formation in mouse models. BET inhibitors can block IGF2BP3-induced NETosis and inhibit NET formation, thereby enhancing the lytic activity of oHSV and improving therapeutic efficacy (211). The formation of NETs may impede the efficacy of DC vaccines by disrupting the antitumor responses induced by NK and T cells. Combining DC vaccines with NET inhibitors could be a promising strategy to enhance antitumor responses (175).

In addition, neutrophil engineering technologies that involve rational modifications of neutrophils have shown promising clinical prospects. For example, neutrophils loaded with paclitaxel (PTX)-loaded liposomes and neutrophil-derived exosome (NEs-Exos) loaded with doxorubicin have demonstrated the ability to effectively penetrate the BBB and target tumors (212, 213). Other therapeutic strategies, including neutrophil-associated nanoparticles (NPs), liposomes, and viral delivery systems, are extensively discussed in another review (214).

## 8 Conclusion

Neutrophils, as an important component of the immune system, are gradually revealing their roles in brain tumors. It has been demonstrated that there is a correlation between neutrophils and gliomas, and future attention should be paid to how the dual role of neutrophils can switch. Efforts are still required to distinguish neutrophils from PMN-MDSCs, N1, and N2 types, as a comprehensive understanding of the molecular targets of different immune cells is crucial for subsequent targeted therapies based on these insights. Current research on neutrophils primarily utilizes murine orthotopic transplant tumor models, but future studies should extend to human gliomas. The types of immune cell infiltrates vary across different grades of gliomas and different genetic types of GBM, necessitating extensive experimentation to identify the trends that lead to specific types of immune cell infiltrates, in order to design effective therapies applicable to various subtypes or cellular states. Some important processes, such as NET formation, metabolic changes, and neutrophil-T cell crosstalk, are also research focuses. The interactions between neutrophils and other immune cells in the TME are still in the early stages of research. Beyond the crosstalk between neutrophils, tumor cells, and immune cells, the connections between neutrophils and non-immune cells remain unexplored. Neutrophils are among the more fragile immune cells, how can they be extracted with minimal damage to surface proteins? Moreover, during the separation process of tissue samples, spatial information about TANs within the tumor is lost. Addressing these technical challenges and reducing costs are also critical. In addition, advancing the progress of neutrophils in gliomas, targeting the recruitment, reprogramming, depletion of neutrophils, and exploring new directions in neutrophil engineering to enhance drug utilization efficiency are important. The application of neutrophils in cancer immunotherapy should be promoted to provide more effective treatment options for cancer patients.

## Author contributions

CS: Writing – original draft. SW: Writing – original draft. ZM: Writing – original draft. JZ: Writing – original draft. ZD: Writing – original draft. YP: Writing – review & editing. GY: Writing – review & editing.
